# IT-adoption and the interaction of task, technology and individuals: a fit framework and a case study

**DOI:** 10.1186/1472-6947-6-3

**Published:** 2006-01-09

**Authors:** Elske Ammenwerth, Carola Iller, Cornelia Mahler

**Affiliations:** 1 Institute for Health Information Systems, UMIT – University for Health Sciences, Medical Informatics and Technology, Hall in Tyrol, Austria; 2 Institute for Educational Science, University of Heidelberg, Germany; 3 Dept. of Psychiatry, University Hospitals of Heidelberg, Germany

## Abstract

**Background:**

Factors of IT adoption have largely been discussed in the literature. However, existing frameworks (such as TAM or TTF) are failing to include one important aspect, the interaction between user and task.

**Method:**

Based on a literature study and a case study, we developed the FITT framework to help analyse the socio-organisational-technical factors that influence IT adoption in a health care setting.

**Results:**

Our FITT framework ("**F**it between **I**ndividuals, **T**ask and **T**echnology") is based on the idea that IT adoption in a clinical environment depends on the fit between the attributes of the individual users (e.g. computer anxiety, motivation), attributes of the technology (e.g. usability, functionality, performance), and attributes of the clinical tasks and processes (e.g. organisation, task complexity). We used this framework in the retrospective analysis of a three-year case study, describing the adoption of a nursing documentation system in various departments in a German University Hospital. We will show how the FITT framework helped analyzing the process of IT adoption during an IT implementation: we were able to describe every found IT adoption problem with regard to the three fit dimensions, and any intervention on the fit can be described with regard to the three objects of the FITT framework (individual, task, technology). We also derive facilitators and barriers to IT adoption of clinical information systems.

**Conclusion:**

This work should support a better understanding of the reasons for IT adoption failures and therefore enable better prepared and more successful IT introduction projects. We will discuss, however, that from a more epistemological point of view, it may be difficult or even impossible to analyse the complex and interacting factors that predict success or failure of IT projects in a socio-technical environment.

## Background

It is hard to imagine health care without Information and Communication Technology (ICT). Information technology in health care has existed for about four decades, and has gained widespread usage. Electronic patient records offer health care professionals access to vast amounts of patient-related information; decision support systems support clinical actions; and knowledge servers allow direct access to state-of-the-art clinical knowledge to support evidence-based medical practice [1].

Introduction of ICT can radically affect health care organisation and health care delivery and outcome. It is evident that the use of modern ICT offers tremendous opportunities to support health care professionals and to increase the efficiency, effectiveness and appropriateness of care [2,3].

However, not all projects introducing IT in health care are successful. It is estimated that up to 60 – 70% of all software projects fail (e.g. [4]), leading to enormous loss of money within healthcare and also to loss of confidence on IT from the side of users and managers.

It is interesting to recognize that the same IT system can be seen as success by one department or professional group, but as a failure or at least as problematic by another department or professional group. Various interconnected factors seem to exist that influence success or failure. In fact, the notion of success and failure has been largely discussed in the literature in the last years. We will not try to repeat the overall discussion here, but just refer to some good references ([5-11]).

What we observe in any case is that the objective effects of the same IT system can largely differ in different settings. This is not surprising if we understand information systems as technical systems embedded in a social-organizational environment (see also [12]). The technology we are introducing in different clinical settings can be largely equal (e.g. the same PACS software in various radiological departments). But the socio-organizational setting may be quite different (e.g. different organization of workflow, different patient profiles, different motivation of staff, different management support, different IT history etc.), leading to different adoption processes of the same IT system, and thus to different effects (e.g. increased efficiency on one ward, user boycott on the other ward).

What does this mean for a systematic IT management in hospitals? We argue that it would be helpful to know more about the factors influencing IT adoption, success and failure, and to be able to predict the effects in a certain setting.

Therefore, at least two questions arise which should be answered by medical informatics research:

1. What are the "socio-organizational" factors that influence adoption of an IT system in a given socio-organizational context?

2. Based on the answers to question 1: Is there any way to predict the effects of an IT system in a certain context?

### The aim of this paper

The aim of this paper is to present an approach to answer the first question. Based on a literature study, we will present a framework (the FITT framework) to better analyse the socio-organisational-technical factors that influence IT adoption. We will present the application of this framework in the analysis of a case study, describing the adoption of a nursing documentation system in several departments of a German University Hospital.

With regard to the second question, we will argue that from some more philosophical point of view, the exact prediction of success and failure may not be possible at all.

### Previous work on IT adoption

Analysis of the factors influencing adoption (and thus also success and failure) of IT systems in health care has been an issue in research for many years. We will define IT adoption as follows, based on the discussion in [13]: for voluntary used system, IT adoption is reflected in the usage of the IT system; for mandatory used systems, IT adoption is reflected in the overall user acceptance. In the next paragraph, we will analyse some research results on factors for IT adoption, focussing on general valid frameworks.

**Figure 1:**
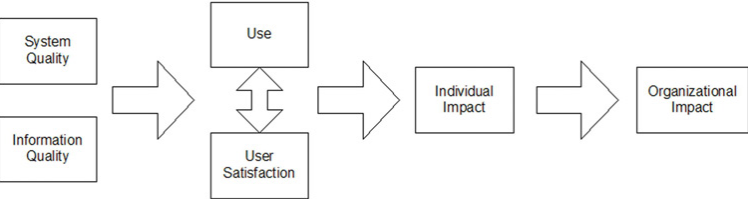
Information success model by DeLone [5].

Analysing the concept of information system (IS) success, DeLone [5] developed an **information success model **for management information systems. This model describes that the effects of IT on the user (the individual impact) and thus on the overall organization depends on the use and the user satisfaction. Those two aspects themselves depend on the quality of the IT system and the quality of the information in this system (Figure 1). This model was used to structure a broad literature review, but seems not to be further validated. The authors discuss that IS success is a multidimensional construct based on the interaction of factors, and that a corresponding measurement instrument should therefore include not only the described criteria, but also their interaction.

The information success model is quite interesting as it describes the *interaction *of various factors. However, its shortcoming seems to be the isolated focus on IT quality and system quality, indicating that only the system's quality itself determines the overall impact. This does not help to explain why the same IT system can be adopted in a different way, and have rather different effects, in various settings.

**Figure 2:**
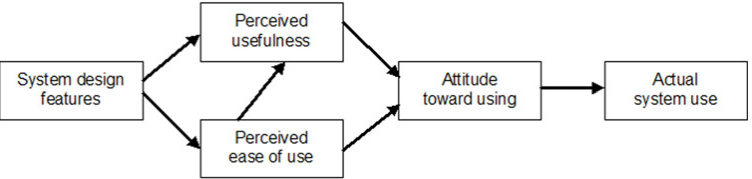
Technology acceptance model (TAM) by Davis [14].

The **technology acceptance model (TAM) **of Davis [14] tries to analyse why users adopt or reject a system. It defines the constructs "perceived ease of use" and "perceived usefulness" to predict attitude towards using and actual system use. Both factors themselves depend on features of the system (Figure 2).

While trying to verify his model by questioning 112 users of one company, Davis [14] could partly confirm the expected links in his model. In his discussion, he stresses that this model is only usable for voluntary use of IT system, and that further factors should be included in his model, such as extrinsic motivation, user experiences with the system, and characteristics of the task to be supported by IT (e.g. complexity of a task).

This TAM model was adopted and extended by other researchers such as [15,16] and [17]. For example, Dixon [16] extended it to the **Information Technology Adoption Model (ITAM)**. He tried to refine the "system design features" of the TAM model by describing that an IT system has requirements (such as required IT knowledge of the users, or necessary technical infrastructure) that must be matched with the knowledge and skills of the users and with the available technical infrastructure. He called this "fit" and argued that perceived usefulness and perceived ease of use are not dependent on the system design features, but on this *fit *of user and system design features. The paper stays unclear whether the ITAM model was more formally validated. It is also unclear why those points already discussed as missing by Davis [14] (such as extrinsic motivation or task characteristics) were not included.

All of the presented models seem to concentrate rather strongly on individual attribute of the users and of the technology, neglecting attributes of the clinical environment and of the supported clinical tasks that in our opinion are of high importance to understand IT adoption processes. ITAM is however interesting as it introduced the *notion of fit*, explaining that it is not individual attributes which are important, but the quality of fit between e.g. IT complexity and IT knowledge.

**Figure 3:**
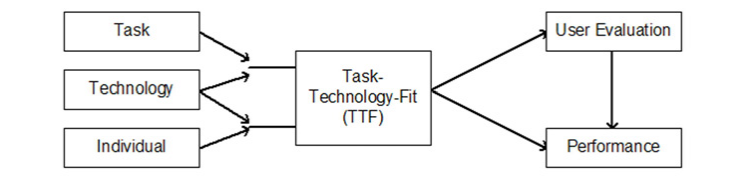
Task-Technology-Fit model (TTF) by Goodhue [8], [13], [18].

The idea of fit is more comprehensively elaborated in the **task-technology-fit model (TTF) **of Goodhue [8,13,18]. He takes into account not only technology and user, but he also considers the complexity of the clinical tasks which have to be supported by an IT system. He examines the influence of the three factors – individual abilities, technology characteristics, and task requirements – on performance and on user evaluation of IT systems, highlighting the significance of the interaction (fit) of those three factors (Figure 3). He argues that TTF (task-technology fit, or more correct task-individual-technology fit, as explained by [13]) is the extent to which technology functionality matches task requirements and individual abilities. Goodhue argues that user evaluation is a sufficient surrogate of TTF, and that it is appropriate for both mandatory and voluntary used IT system. The TTF model was used in the area of management information systems, and many of the proposed links within the model could be validated in studies in various studies with hundreds of users.

TTF extends the other described models by concentrating on the fit. IT also includes the object of clinical task (e.g. task complexity, organization of tasks, interdependence with other tasks) to be supported by IT. However, TTF only focuses on the fit between user and technology, and between task and technology (see Figure 3). It does not consider the interaction of user and task – which is, however, in our opinion an important success factor for IT introduction projects. For example, introduction projects may fail because nurses are not sufficiently motivated for nursing process documentation at all, independent of the tool used, or physicians may not be motivated to do a complete order entry themselves, instead of ordering a nurse to complete the order, because of the additional time it will take them. In addition, TTF and derived models do not reflect on the dynamics of introduction projects. Attributes of users, task and technology frequently change over time in a clinical environment, and thus also their interaction and their fit change.

However, the notion of fit has been found useful in many other studies, too. For example, Folz-Murphy [19] described problems of the fit between user requirements and available IT functionality. Zigurs [20] examined the fit between task and technology in the area of group supports systems. Dishaw et.al. [21] extended the TTF – combined with the TAM model – with the construct of computer self-efficacy. With reference to the domain specific of users abilities the developed model of Dishaw et.al. also implied a relation between the attributes of user and task. The idea of fit seems thus to be helpful in various contexts.

Overall, the presented approaches present a good basis for the analysis of the IT adoption; however, all of them show some limitations.

Bases on this analysis of the literature, we will now present a framework of fit between individuals, task and technology (FITT framework), taking into account the process-oriented character of an IT introduction. We will use our framework in a retrospective analysis of a corresponding case study.

## Methods: The FITT framework

Based on the literature review, we found it useful to use the interaction (fit) of users, tasks and technology as the basis to better understand IT adoptions.

**Figure 4:**
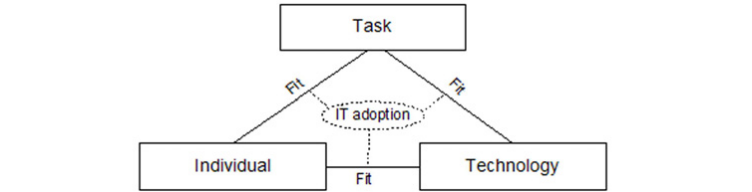
The FITT framework (1): IT-adoption depends on the fit between individual, task and technology.

Our FITT framework ("**F**it between **I**ndividuals, **T**ask and **T**echnology") is based on the idea that IT adoption in a clinical environment depends on the fit between the attributes of the users (e.g. computer anxiety, motivation), of the attributes of the technology (e.g. usability, functionality, performance), and of the attributes of the clinical tasks and processes (e.g. organisation, task complexity) (Figure 4).

An "Individual" can represent an individual user or a user group. "Technology" can stand for the interaction of various tools needed to accomplish a given tasks (e.g. hardware, software, network). But the technology does not only comprise computer-based tools, but all tools used by the individuals to execute the tasks, therefore including also paper-based tools. "Task" comprises the wholeness of tasks and working processes that have to be completed (e.g. nursing documentation, order entry etc.) by the user and that are supported by the given technology.

Many researchers focus on the aspect of "organisation". Organisational aspects in our model are either part of the individual aspect (individuals work in various roles and various groups in an organization), or they are considered in the task aspect (the clinical tasks and processes are organized in a given way, with defined responsibilities).

The objective of IT management can now be defined as reaching an optimal fit between technology, user and task. This means that e.g. user involvement in the selection process or a good user support can improve the fit between the three aspects. Individuals must therefore be *sufficiently *motivated and knowledgeable to execute a certain task. The technology must offer *sufficient *functionality and performance to support a given clinical task. And the user must be *sufficiently *trained to use a given technology adequately. An insufficient fit will probably lead to problems during implementation projects.

The **quality of fit **depends on the attributes of the objects. The following list presents some examples on attributes that affect the various fit dimensions:

• Attributes on individual level: IT knowledge, motivation and interest in the task to be completed, flexibility and openness to new ways of working, team culture, organizational context, cooperation within a team, and politics within an organisation.

• Attributes on task level: Organisation of the tasks to be completed, activities and their interdependence, complexity of tasks.

• Attributes on technology level: Stability and usability of a software or hardware tool, costs of a tool, functionality, available technical infrastructure, integration of tools, availability of tools in a certain clinical situation.

In order to influence and improve the fit, management can *directly influence *those attributes of task, individual, and technology. For example, a reorganization of documentation processes may improve the fit between task and technology; training sessions for users may improve the fit between technology and individuals; a software update may influence both the fit between technology and task (e.g. new functionality being implemented) and between individual and technology (e.g. usability being improved). Here are some examples for possible deliberate **interventions **on the three objects to influence and optimize the fit:

• Intervention on the individual level: user involvement in system selection and introduction (change management), user training sessions, good user support, motivation by the management (leadership issues).

• Intervention on the task level: Reorganisation of task and working processes (e.g. new ways for order entry), clarification of the responsibilities (e.g. for nursing documentation).

• Intervention on the technology level: Hardware and software updates, redesign of paper-based forms, network upgrade.

Besides the direct interventions on the three objects, there are also external factors that may influence the fit, but which cannot easily be controlled by the IT management. The following list presents examples for those **external influencing factors**:

• Intervention on the individual level: Staff changes (e.g. reducing IT knowledge), workload of staff (e.g. reducing time for IT use), changes of hospital strategy (e.g. IT is now seen to contribute to competitiveness of the hospital).

• Intervention on the task level: Rising complexity of the task (e.g. by new legal documentation requirements), general organisational changes in the organisation, changes in patient profiles.

• Intervention on the technology level: New software standards, new technological achievements.

**Figure 5:**
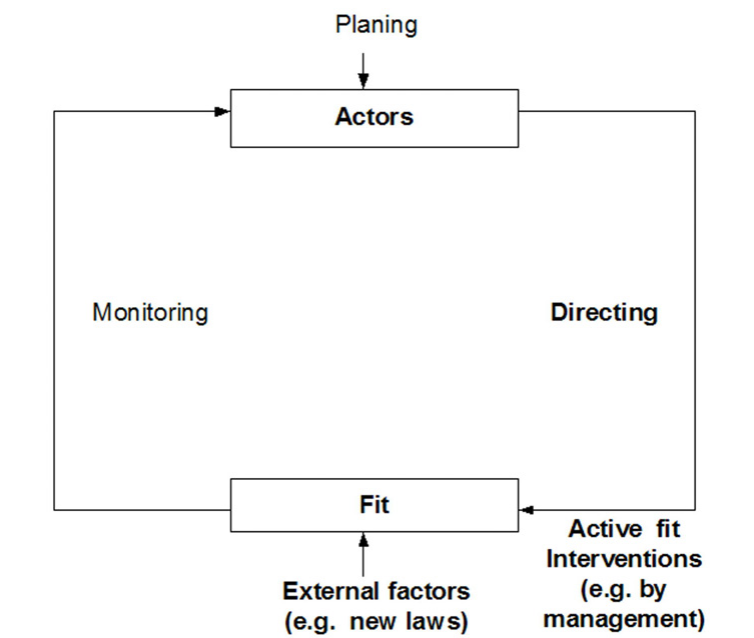
Planning, directing and assessment of the fit. While the fit can be managed by deliberate active interventions (e.g. by IT management), continuous external factors may influence it, too.

Due to those external factors, there will never be a complete static situation with regard to the three fit dimension and therefore to IT adoption. The external factors can improve or deteriorate the fit, while the deliberate interventions of IT management will be aimed at steadily improving the fit. There may only be a partly stable situation where the positive and negative changes are mostly balanced. It is helpful to describe this fit management and fit dynamics as a **loop-back system **(Figure 5).

The overall aim is to have an optimal fit to allow an easy IT adoption. As described, the fit model allows us to describe what we can do to influence and balance the fit. The larger the difference between the actual fit and the planned fit, the higher the problems during an IT introduction. For example, low fit between users and technology may lead to user frustration and finally to user boycott if no interventions (e.g. IT training sessions) are organized.

**Figure 6:**
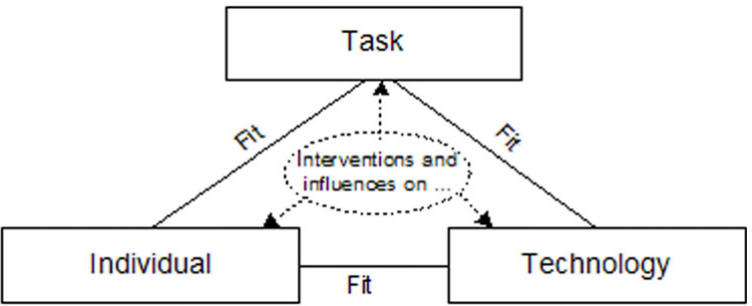
The FITT framework (2): Deliberate interventions and external influences will affect attributes of task, technology and fit, thereby indirectly affecting the three fit dimensions.

We assume that this basic theoretical approach can help analyzing the process of IT adoption during an IT implementation project in a clinical environment in the following ways (Figure 6):

1. Any disruptions during an introduction project can be described and analysed with regard to the disruption in one of the **three fit dimensions **(task-technology, technology-individual, or individual-task). This should help plan projects, as problems can be anticipated in advance, or can help to analyse problems in a project retrospectively in order to learn from them.

2. Any intervention that is taken to improve a project, to make it successful, can be analysed and described with regard to one of the **three objects **(task, individual, or technology). Any of those interventions on the objects will thereby indirectly affect the fit dimensions.

We will now present a case study where the FITT framework was applied in a retrospective analysis, to show how it can help describe and analyse an implementation project.

## Reanalysis of a case study: IT adoption and FITT framework in a German university hospital

A computer-based nursing process documentation system was introduced on several wards of the University Hospitals of Heidelberg between 1998 and 2001. This introduction was accompanied by various evaluation activities which among others investigated the following aspects:

• General computer knowledge and attitudes to computers in nursing before, during and after system introduction.

• Nurses acceptance of the nursing care process (the task to be supported by the IT) before, during and after system introduction.

• User satisfaction with the nursing documentation system before, during and after introduction.

• Quality of nursing documentation before, during and after system introduction.

• Overall affects of the nursing documentation systems on nursing workflow.

These evaluations were done e.g. based on standardized and validated psychometric questionnaires (given to all nurses, with return rates around 80%), standardized documentation quality audits (analysing the nursing records of 20 patients per ward at three points of time), and focus group interviews with 1 – 2 nurses per ward and with nursing and project management. Methods and results of the evaluation studies have been published e.g. in [22-25]. More details on all studies can be found in the corresponding German research reports [26-28] as well as in [29].

**Figure 7:**
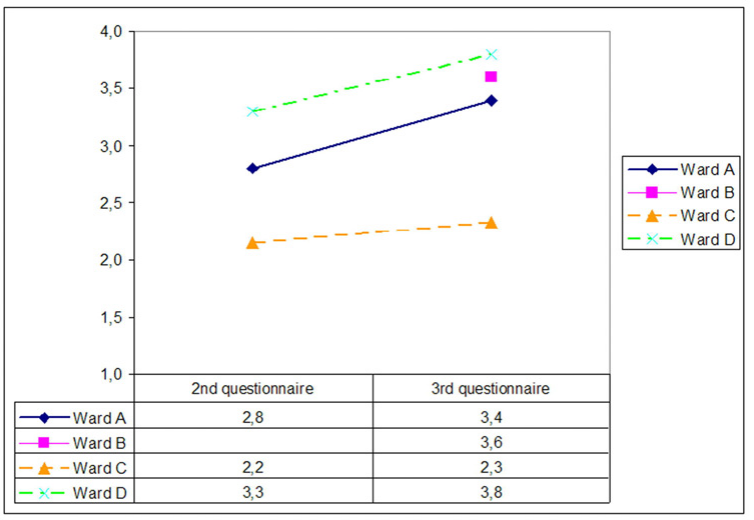
Answer to the question "Do you want to continue working with the nursing documentation system" on four wards (n = 56 for all 4 wards; 1 = no, 4 = yes; indicated is the mean of all answers). The 2nd questionnaire was applied around 3 month after IT introduction (except Ward B), the 3rd questionnaire at least 6 months after the 2nd.

In general, the evaluation results showed high user acceptance of the IT system, and positive effects e.g. on documentation quality. A detailed analysis, however, showed differences in the reactions of the wards with regard to the new IT system. On one (somatic) ward, user acceptance was much lower than on the other wards, and several problems during IT introduction occurred here. On this ward (ward C), user acceptance was very low shortly after the introduction, and remained rather low even months after it (Figure 7).

The FITT framework was used to analyse the differences on the wards, the process of IT adoption, and the effects of interventions taken by the project and IT management to improve IT adoption. This analysis was based on the available results of the already mentioned various specific evaluation studies.

In this paragraph, we will present the result of this analysis for two somatic and two psychiatric wards. As already discussed, three of them showed a quick IT adoption, one of them showed a more problematic introduction (Figure 7). A complete report of this analysis has been published in a German project report [27].

All wards had used a paper-based documentation system prior to IT introduction which was now in part replaced by a computer-based system. This new IT system covered all steps of the nursing process (nursing anamnesis, care planning, documentation and evaluation of care – for a detailed explanation of the nursing process, see e.g. [30]). However, all functionalities were only used on the psychiatric wards where all steps of the nursing process were documented. The documentation on the somatic wards concentrated on the documentation of nursing anamnesis, care planning, nursing tasks, and omitting the evaluation of care. Nursing notes were written on all wards in the IT system.

### Dermatological ward

The dermatological ward had 20 beds, around 12 nurses and a mean length of stay of about 10 days in 2000. The IT system was introduced in Sept. 2000. Questionnaires and documentation analysis were conducted three months before IT introduction and again in Dec. 2000 and in June 2001. A focus group interview study was conducted in February 2002.

The analysis on this ward found a rather uncomplicated and quick adoption of the new IT system. We will present the reanalysis of this case on the three fit dimensions:

• **Fit between individuals and task**: This fit was mostly uncomplicated from the very beginning. Both ward managers and nurses stated in the interviews that they were convinced of the necessity of a high-quality nursing documentation, for legal reasons and for the reputation of nursing. The nursing process was mostly well accepted, as the questionnaires showed. Documentation analysis and interviews confirmed that nursing documentation was more complete after IT introduction than before. However, as the intensive documentation audits showed, not all steps of the nursing process were well documented, and the documentation was in part not adequately adapted to the individual patient.

• **Fit between individuals and technology**: This fit was uncomplicated from the very beginning. The young, motivated team with high IT skills had no problems in learning the new technology. Computer acceptance and computer security levels were found to be high from the very beginning both in the questionnaires and the interviews.

• **Fit between task and technology**: This fit was a bit problematic at the beginning, as the documentation analysis showed. The pre-defined nursing care plans offered by the IT system were at first not sufficiently adapted to the need of this ward. In addition, the computer equipment was first insufficient (to small number of computers, too slow hardware) to support a timely documentation process. Because documentation has always been done in the ward headquarters, no mobile or bedside computers were found necessary.

**Figure 8:**
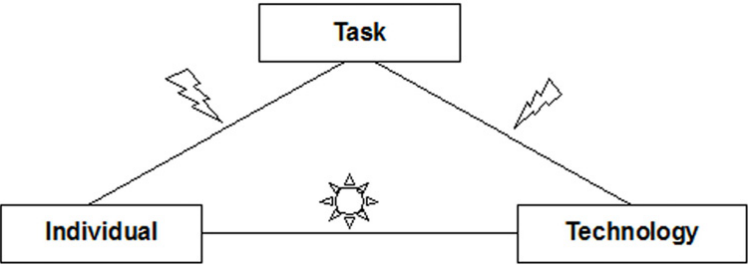
Analysis of the Fit on a dermatologic ward shortly after introduction of the computer-based documentation system. An arrow indicates problems with the fit, a sun indicates an uncomplicated fit.

Summarizing, on the dermatologic ward, we found a good individual-technology fit after the IT introduction. The individual-task as well as the task-technology fit were not optimal at the very beginning (Figure 8).

In order to improve the problematic fit dimensions, project management intervened as follows during the introduction period:

• **Intervention with regard to task**: None.

• **Intervention with regard to user**: Several onsite discussion to increase nurses' knowledge of the nursing process and how to correctly use pre-defined standardized nursing care plans, to increase fit between individual and task.

• **Intervention with regard to technology**: The pre-defined standardized nursing care plans were refined, to improve adaptation to the individual patient; hardware was updated and extended, thereby increasing fit between task and technology.

Those interventions seem to have improved the fit. The nurses judge the support of documentation by the software and hardware equipment as rather good after two years both in the interviews as well in the standardized questionnaires. The documentation analysis also show an improvement in documentation quality.

### Paediatric wards

The paediatric ward had 15 beds, around 13 nurses, and a mean length of stay about 5 days in 2000. The nursing documentation system was introduced in Oct. 2000. Questionnaires and documentation analysis were conducted three months before IT introduction and again in Jan. 2001 and in July 2001. A focus group interview study was conducted in February 2002.

Compared to the other wards this ward showed rather low user satisfaction values with the nursing documentation system during the introduction phase. An analysis structured according to the FITT framework showed several problematic areas:

• **Fit between individuals and task**: The detailed documentation audits showed that nursing documentation was incomplete both before and after IT introduction (for details, see [24]). The documentation audits showed that the amount of documentation rose heavily during IT introduction, but documentation quality did not increase in the same manner (e.g. inadequate adoption of standardized nursing care plans to the individual patient). User attitudes with regard to the nursing care process strongly declined after IT introduction (details e.g. in [23]). In questionnaires and interviews, users complained about high time efforts for documentation. These and other results indicated that the fit between individuals and task may have already been problematic before IT introduction, and now deteriorated after IT introduction, as the new IT tool forced a more complete documentation, without bringing obvious benefits to the nurses.

• **Fit between individuals and technology**: Validated questionnaires as well as focus group interviews showed some initial problems handling the new hardware and software. As the questionnaires showed, the users were rather unfamiliar with computers in the beginning. Some of the users were not too enthusiastic to learn the new IT system. However, the general attitudes with regard to computers in nursing were comparable to the other wards and on a medium level at the beginning. All in all, there were only some smaller problems in this fit on this dimension.

• **Fit between task and technology**: This fit was found to be very problematic. Focus group interviews with users and managers revealed that in the beginning the software was not optimally customized. For example, the predefined nursing care plans in the software were found to be insufficiently adapted to the patients of this ward (a problem comparable to the dermatological ward). Also, the functionality and performance of the system was judged to be insufficient in some parts. For example, the repeated documentation of one item during a longer time period was not well supported. A big problem was also that no mobile computers or bedside terminals were available, which disturbed the common documentation workflow – while the nurses on this ward were used to documenting at least some aspects at the patients bedside, this had not been reflected by adequate hardware equipment in the introduction phase. From the users point of view, this all led to high and unnecessary time efforts for documentation.

**Figure 9:**
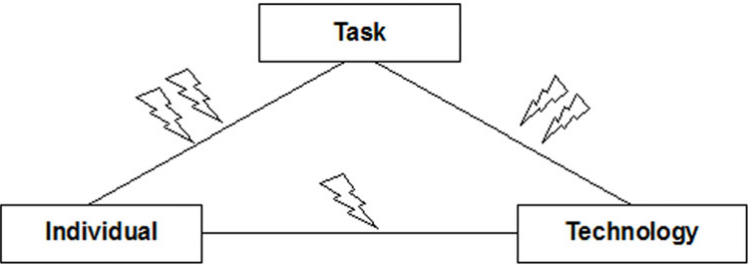
Analysis of the Fit on a paediatric ward shortly after introduction of the computer-based documentation system. One arrow indicates smaller problems with the fit, two arrows larger problems.

Summarizing, on this ward, all three fit dimensions were disturbed in the introduction period (Figure 9). Therefore it is not surprising that we found rather low user satisfaction (e.g., about half of the users wanted to stop using the software after three months) during this period.

Due to these problems, project management decided on the following interventions which we have structured according to the FITT framework:

• **Intervention with regard to task**: The workflow for documentation was reorganized, e.g. the number of items which needed to be documented were reduced, and some intermediate paper-based documentation was allowed to react on the missing mobile tools. This improved the individual-task as well as the task-technology fit.

• **Intervention with regard to user**: Onsite training to refresh knowledge on nursing process and nursing documentation helped to increase the individual-task fit. Further individual training sessions with regard to computers in general and the software were organized. This helped increase the fit between individual and technology.

• **Intervention with regard to the technology**: Missing functionality was implemented, erroneous functions were corrected, and hardware was updated to increase the performance, thus increasing the fit between task and technology.

All those interventions affected the three fit dimensions differently. The repetition of the quantitative evaluation about 9 months after implementation indicated a clear improvement in user satisfaction, reflecting in our opinion an improvement in the fit, which was also supported by the interview study. In addition, in the documentation analysis, the amount of documentation was now found to be reduced.

### Psychiatric wards

As both psychiatric wards were found to be rather similar in IT adoption, they will be discussed here together. The wards had 21 resp. 28 beds and around 19 resp. 17 nurses. Mean length of stay was around 21 resp. 14 days in 2000. The nursing documentation system was introduced in Nov. 1998 resp. Nov. 1999. Questionnaires and documentation analysis were conducted three months before IT introduction, in Febr. 99 resp. March 2000, and again in Aug 2000. A focus group interview study was conducted in February 2002 with nurses from both wards. Both wards had long-term experience with paper-based documentation of the nursing process.

Both wards showed a mostly uncomplicated IT adoption:

• **Fit between individuals and task**: This fit was uncomplicated from the very beginning. Nursing documentation and nursing process were highly accepted by ward management and nurses, as reflected in the questionnaires and interviews. Documentation analysis found high quality and completeness of documentation, even when some parts still appeared to be too standardized.

• **Fit between individuals and technology**: Nurses were motivated to work with the new system. At the beginning, some nurses were not very IT experienced and had some initial problems, but computer acceptance scores were nevertheless high. User confidence and security in working with the IT system was found to be rather high.

• **Fit between task and technology**: In the beginning, performance and functionality of the IT system were regarded as insufficient by the nurses. Also the quality of the predefined nursing care plans were found weak. Nurses felt that the system was not very useful to support nursing documentation in the first months.

**Figure 10:**
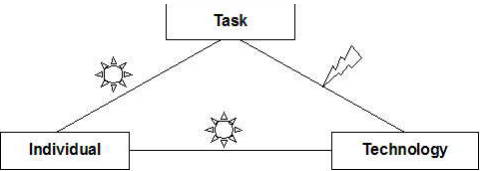
Analysis of the Fit on two psychiatric wards shortly after introduction of the computer-based documentation system. An arrow indicates problems with the fit; a sun indicates an uncomplicated fit.

Summarizing, on these wards, the fit dimensions were rather good, with some problems only in the fit between task and technology (this comparable to the other wards) (Figure 10).

Project management decided on the following interventions that we have structured according to the FITT framework:

• **Intervention with regard to task**: None.

• **Intervention with regard to user**: Some individual computer support was offered to increase fit between technology and individual.

• **Intervention with regard to the technology**: Missing functionality was implemented, erroneous functions were corrected, and hardware was updated to increase the performance, therefore increasing the fit between task and technology.

These interventions helped to optimize the fit. The evaluations after several months and even years after implementation showed high user satisfaction and an improvement in nursing documentation quality, although some functions of the system were still being criticised for not being adequately adapted to the specific needs of a psychiatric ward.

## Results: Facilitators and barriers to IT adoption

Based on the result of the analysis of our different study wards, we will now collect the factors that seem to represent facilitators and barriers to adoption of a computer-based nursing documentation system. Based on the assumption that IT adoption depends on the fit between individual, task and technology, we found indicators in the reanalysis of our case study that affect IT adoption of nursing documentation systems (formulated in the way that the "higher/better" the attribute, the easier IT adoption):

• Relevant attributes of **individuals**: Commitment to nursing process as basis for nursing, commitment to nursing care planning, commitment to written nursing documentation, commitment to own professional nursing role (IT as professional tool), acceptance of computers in general, acceptance of computers in nursing, computer skills, typing skills (may be correlated with computers skills), general computer knowledge in years, age of nurses (may be correlated with computer knowledge), professional experience (may be correlated with age), number and motivation of key-users, overall motivation of wards to introduce the system, climate of support and trust within the nursing team, quality management skills of nurses, low expectations with regard to computers and nursing documentation, low number of staff members and work load of ward, low staff fluctuation, low number of part-time staff, night watches and nursing trainees on the ward, commitment to standardisation of nursing tasks (IT as support, or IT reducing individuality of nursing).

• Relevant attributes of the **task of nursing documentation**: Low complexity, amount and level of detail of documentation, clear organization, clearly structured place and time of documentation, quality of implemented predefined nursing care plans, low number of nursing tasks that have to be documented in each shift, low use of documentation (e.g. once per shift), long length of stay of patients, low complexity of patient profiles (children, adults), high use of documentation by other health care professionals, available time during routine work to learn the system, no parallel redundant use of different documentation media (IT, paper), clear agreements with regard to organisation of documentation, availability of nursing standards from other wards or earlier projects, high degree of standardisation of nursing.

• Relevant attributes of the **technology**: Quality and amount of functionality of software, usability and user friendliness of software, stability and flexibility of software, quality and performance of hardware and network, availability of sufficient number of computers, availability of mobile computers, clear version and update management.

Based on our analysis, the following interventions and external factors can be found which may have a positive influence on the fit between individuals, technology and tasks (mostly corresponding to the "active interventions" in Figure 5) and therefore on IT adoption:

• Positively affecting **individual-technology fit**: IT training sessions, positive external norms (e.g. computers belong to nursing), high computer acceptance by nursing management, high motivation and training of key users, intensive user support, step-wise implementation of functionality (instead of all at one point of time), reduction of nursing workload during the introduction phase (e.g. by additional staff).

• Positively affecting **individual-task fit**: Efficient training sessions on the nursing process, high acceptance of nursing process by nursing management, high external norms (e.g. nursing is an own profession), clarification of responsibilities within nursing documentation, reorganization and restructuring of nursing documentation processes, clarification of the sensible amount of nursing documentation (to avoid over-documentation), increased use of predefined nursing care plans, step-wise introduction of nursing process.

• Positively affecting **task-technology fit**: Reorganisation and restructuring of nursing documentation, local adaptation of predefined nursing care plans, update of software functionality enabling the reflection of the tasks characteristics for a ward, increase in number and availability of computers, introduction of mobile tools in case the tasks make this necessary.

Please note that the interventions do in fact directly influence attributes of individual, technology, or task, thereby only indirectly influencing one or two of the three fit dimensions. For example, by organizing additional training session, we can improve the IT skills (attributes) of the individuals, and thereby also indirectly influence the individual-technology fit.

This analysis should highlight that – even for this rather restricted case study and the limited focus on nursing documentation – a variety of factors can be found that influence the fit between individuals, technology and task and therefore IT adoption. This supports the often discussed fact that success and failure is a rather complex and multi-dimensional construct.

## Discussion

In this paper, we presented – based on an analysis of other IT adoption frameworks from the literature – a framework of the interaction between individual, technology and tasks (the FITT framework). This framework was used to support a structured retrospective analysis of the introduction of a nursing documentation system in a German University Hospital. The detailed analysis of the case study showed common features, but also differences of IT adoption of the wards which could be easily reflected and analysed based on the FITT framework.

The FITT framework focuses on the significance of the optimal interaction (fit) of individual user, technology, and task. The fit between the attributes is more important than the individual attributes themselves. For example, IT skills of the users are not sufficient for the success of an introduction – rather, they must match the requirements by the IT software (e.g. software complexity).

In our case study, the clear structure with three objects and three fit dimensions helped us reflect on the different reactions of the wards we had found in the evaluation studies, on the problems which occurred during introduction, and on the interventions of project management. We did not find any aspect that we could not easily structure within this FITT framework. This however can not be regarded as formal proof of completeness of our framework.

The idea of fit has been introduced by other authors before, such as [16] or [18]. Nancy Levensen discussed in here keynote at the Information Technology in Health Care Conference (ITHC 2004) in Portland that system failure often depends on failures in the interaction between components, not on the quality of the components themselves that often do not present problems from an isolated point of view. Southon [31] discussed the fit between organization, technology and user skills. Lundberg [32] examined the interaction between actors (staff), artefacts (technology) and working processes during a PACS installation. And Palvia [33] analysed the significance of the factors task, technologies, user, and organisation during a system introduction.

But none of those previous and the other analysed authors, to our knowledge, noticed the important interaction between user and task. There are many examples e.g. in the area of nursing documentation systems or computerized physician order entry where the users were not motivated to do a certain task – independent of the quality and functionality of the IT tool that was introduced! The reason why this interaction is often overlooked seems simple: In many cases, IT introduction is accompanied by organizational changes (e.g., when CPOE is introduced, a much higher documentation burden is suddenly put on the physicians), often leading to low user satisfaction or even user boycott (see example in [34]). These problems are then often attributed to the IT system (suspecting a low fit between IT and user or between IT and task). But in fact the problems are mostly coming from a more fundamental ill-acceptance of the new task to be done, thus reflecting a low fit between user and task! For an example of this ill-acceptance, see the detailed analysis of a CPOE introduction by Massaro [35].

Management of fit can be regarded as a loop back system, reflecting that the fit is never really stable, but that it is changing based on external factors or deliberate interventions, making fit management a constant and complex task for the whole life cycle of an IT system.

In our case, all wards showed specific and time-dependent IT adoption processes while starting to work with computer-based nursing documentation system. Unfortunately, we could not analyse this process in detail, as the measurement points of the various studies were too different and irregular (the available data came from four partly independent evaluation studies). A more refined analysis would have needed an analysis in regular short intervals. Thus, all our presentations with regard to the dynamic of the IT adoption within the wards must be taken with care – e.g., in Figure 7, we do not know whether there have not been ups or down of user satisfaction in between the two measurement points.

All wards are now working rather successfully with the computer-based nursing documentation system, however, still the fit is not in complete balance, as further changes are steadily occurring (e.g. new staff members need to be trained, new documentation standards have to be implemented, software errors lead to updates, refinement of organization of nursing documentation, new documentation guidelines leading to training session etc.). We expect that managing the fit balance is a continuous task which is never really completed, and can be described as a loop-back system (cp. Figure 5).

Our case study shows the complexity of a broad introduction of an IT system in various settings: Individuals and tasks are rather different in the various settings, requiring high flexibility of the IT system and individual IT introduction and support activities to get the best fit for each ward. This helps to explain why we can find different IT adoption even when the same software and hardware is introduced.

Interestingly, it seems that the involved users often are not able to distinguish between the various fit dimensions. For example, the nurses in our study partly expressed the opinion that the new software system was not usable. Detailed analysis revealed that at least some problems were based on the miss-fit between user and task – and not on a miss-fit between user and technology.

In this context, we want to stress the significance of the user in IT introduction projects. Investing in user training and user support can have positive effects on both individual-task and individual-technology fit. In addition, user involvement in system design and selection helps to build more adequate systems, therefore also improving the task-technology fit. And, as Goodhue [8] shows, user evaluation is a good surrogate for the overall fit, explaining partly why user acceptance studies have found so widespread use (which should not be understood as to underestimate the significance of objective performance measures).

In case we accept the FITT framework as a point of basic understanding of IT adoption – what are the following steps in ongoing development of this framework? Some may argue that the first logical step would now be to develop measurement instruments for the fit (as e.g. Goodhue [18] tries for his task-technology fit) which in fact seems necessary. The second step could then be to quantify the factors influencing the fit, to allow better and quantifiable prediction and planning of successful IT adoption – e.g. if we knew that IT skills explain around 65% of the variability of fit between IT and users, then we may consider it useful to invest in training session. Comparable quantitative oriented research on factors was e.g. done by [23,36] or [37]. To achieve this goal, we would have to compile a complete list of attributes of tasks, technology and individual influencing the fit. However, this research approach would only make sense from a realistic (or positivistic) point of view where we expect that an absolute reality exists, where objects have attributes which we can unambiguously measured.

From another perspective that is often called relativistic or constructivistic, this approach may be seen as misleading, as no absolute and measurable reality is thought to exist. In any case, we are dealing here with people whose reactions to given inputs cannot be precisely predicted – as von Förster [38] would put it, socio-technical systems are non-trivial systems. From this point of view, the significance of the various factors influencing the fit can only be analysed based on the background of a given setting, with all of the political, organizational and individual history influencing the fit. The isolated analysis of say four factors (out of an unknown but probably very large number of existing factors) can never lead to a significant and comprehensive insight into what is going on in a given context. And a generalisation of interactions between these limited numbers of factors could never reflect all possible settings and would thus not be useful. Summarizing, from a more epistemological point of view, it may be difficult or even impossible to analyse the complex and interacting factors that influence the fit.

Without adopting any specific research paradigm here, we would like to argue that research on factors influencing the fit (or IT adoption in general) must try to generalize from individual cases. We must come to a valid measurement instrument for the fit dimensions. And we are convinced that independent of the setting, there may be some priority of factors (e.g. in our case, we found IT skills to be less important than acceptance of the nursing process on all wards). Even such a rough priority list would help IT managers to optimize planning and directing of IT systems in clinical context – without trying to quantify the individual impacts and their interactions, which in fact does not seem helpful. Research in this direction has been done e.g. by [39] or [40].

In any case, at the moment, the FITT framework presents a straight-forward analytic framework to describe and analyse IT adoption case studies. It is innovative in the sense that it clearly describes the objects and their interactions affecting the fit, understanding fit management as a loop-back system.

The FITT framework was based on the retrospective analysis of the adoption of a nursing documentation system on four wards over 3 years. With regard to the broad literature we have discussed in the beginning, showing the usefulness of the notion of fit in various settings, we expect the FITT framework to be valid also for other IT types in other settings – but this still needs to be verified.

The framework should now continue to be refined and also balanced to other adoption theories. And, as said, we need a valid quantitative measurement instrument for the three fit dimensions. The description of the dynamics of change can be improved by introducing a time axis into the framework. After further refinement and validation of this theoretical approach, we expect an even better support for the planning and evaluation of IT introduction projects.

## Conclusion

In this paper, we presented – based on an analysis of other IT adoption frameworks from the literature – a framework of the interaction between individual, technology and tasks (the FITT framework). This framework was successfully used to support a structured retrospective analysis of the introduction of a nursing documentation system in a German University Hospital. Based on this case study, we derived facilitators and barriers to IT adoption of clinical information systems. This work should support a better understanding of the reasons for IT introduction failures and therefore enable a better prepared and more successful IT introduction projects.

## Competing interests

The author(s) declare that they have no competing interests.

## Authors' contributions

The case study was planned and executed by EA. CI participated in the qualitative part of the case study, CM in the quantitative part of the study. The FITT framework was developed by EA and CI together. The paper was written by EA with the support of CI and CM.

## Pre-publication history

The pre-publication history for this paper can be accessed here:


